# Rheumatoid factor isotypes in relation to antibodies against citrullinated peptides and carbamylated proteins before the onset of rheumatoid arthritis

**DOI:** 10.1186/s13075-016-0940-2

**Published:** 2016-02-09

**Authors:** Mikael Brink, Monika Hansson, Linda Mathsson-Alm, Priyantha Wijayatunga, Marije K. Verheul, Leendert A. Trouw, Rikard Holmdahl, Johan Rönnelid, Lars Klareskog, Solbritt Rantapää-Dahlqvist

**Affiliations:** Division of Rheumatology, Department of Public Health and Clinical Medicine, Umeå University, SE-90185 Umeå, Sweden; Rheumatology Unit, Department of Medicine, Karolinska Institute, Stockholm, Sweden; Department of Immunology, Genetics and Pathology, Uppsala University, Uppsala, Sweden; Thermo Fisher Scientific, Uppsala, Sweden; Department of Statistics, Umeå University, Umeå, Sweden; Department of Rheumatology, Leiden University Medical Centre, Leiden, The Netherlands; Medical Inflammation Research, Medical Biophysics and Biochemistry, Karolinska Institute, Solna, Stockholm Sweden

**Keywords:** Rheumatoid arthritis, Rheumatoid factor, Anti-citrullinated peptide antibodies, Pre-symptomatic individuals, Anti-carbamylated protein antibodies, Anti-CCP2 antibodies

## Abstract

**Background:**

The presence of rheumatoid factor (RF), anti-carbamylated protein antibodies (anti-CarP) and antibodies against citrullinated protein and peptides (ACPA) precedes the onset of symptoms of rheumatoid arthritis (RA) by several years. Relationships between the development of these antibodies are not obvious.

**Methods:**

Three isotypes [immunoglobulin A (IgA), IgG and IgM) of RF were analysed in 321 pre-symptomatic individuals who provided 598 samples collected a median of 6.2 (interquartile range 7.2) years before the onset of symptoms, and in 492 population control subjects. All samples were donated to the Biobank of Northern Sweden. RF isotypes were analysed using the EliA system (Phadia GmbH, Freiburg, Germany) with 96 % specificity according to receiver operating characteristic curves. Ten ACPA specificities were analysed using the ImmunoCAP ISAC system, and anti-CCP2 and anti-CarP antibodies were evaluated using enzyme-linked immunosorbent assays.

**Results:**

The frequencies of RF isotypes in pre-symptomatic individuals were significantly increased compared with control subjects (*p* < 0.0001). In samples collected ≥15 years before the onset of symptoms, the IgA-RF isotype was significantly more prevalent than the most frequent ACPAs. Combinations of IgM- and IgA-RF isotypes with ACPA specificities [α-enolase (CEP-1/Eno_5–21_)], fibrinogen (Fib)β_36–52_, Fibα_580–600_, filaggrin (CCP-1/Fil_307–324_) and anti-CCP2 antibodies were associated with a significantly shorter time to onset of symptoms (*p* < 0.001–0.05). Using conditional inference tree analysis, anti-CCP2 in combination with anti-filaggrin antibodies gave the highest probability, 97.5 %, for disease development.

**Conclusions:**

RF isotypes predicted the development of RA, particularly in combination with ACPA, anti-CCP2 or anti-CarP antibodies. The highest probability for disease development was the presence of anti-CCP2 and anti-filaggrin antibodies.

**Electronic supplementary material:**

The online version of this article (doi:10.1186/s13075-016-0940-2) contains supplementary material, which is available to authorized users.

## Background

It has been known for more than half a century that antibodies against the Fc portion of immunoglobulins [rheumatoid factor (RF)] are associated with rheumatoid arthritis (RA) and to a lesser extent with other inflammatory diseases [1–3]. The appearance of RF of the immunoglobulin M (IgM) isotype years before the onset of disease was first reported by Aho and colleagues [4] and has been confirmed in a number of studies showing that RF of the IgM isotype, as well as the immunoglobulin G (IgG) and immunoglobulin A (IgA) isotypes, preceded the onset of RA [4–7]. Anti-citrullinated protein and peptide antibodies (ACPA), also present in sera and/or plasma years before disease onset, have been associated with the development of RA and with a higher diagnostic specificity for RA than RF [6–8]. RF isotypes in combination with anti-cyclic citrullinated peptide 2 (anti-CCP2) antibodies yielded higher risk ratios for disease development than each factor separately, suggestive of an interaction between RFs and ACPA [5, 7]. Multiplex analyses of ACPA in blood samples collected before the onset of symptoms of RA revealed particularly increased concentrations of antibodies against fibrinogen (Fib)β_36–52_, α-enolase (CEP-1) and filaggrin. The frequencies of these antibodies increased gradually, reaching the highest levels just before the onset of symptoms and increasing further after disease onset [9]. Anti-carbamylated protein (anti-CarP) antibodies, another group of antibodies resulting from post-translational modifications of proteins [10, 11], have been shown to be associated with the development of RA, and with radiographic progression, particularly in anti-CCP2–negative individuals [10, 12]. Combinations of antibodies against CarP and CCP2 and/or RF were associated with a modest increased sensitivity for RA, albeit with a decreased specificity [13]. The actual timing of the first antibody to be detectable (i.e., whether it is ACPA or RF) is still uncertain [5, 7, 8, 14]. The results of a number of studies have suggested that RF is produced before ACPA; however, the opposite finding has also been reported [6, 8, 13, 14]. An interesting hypothesis was recently proposed by two different groups of researchers who studied IgM-RF– and ACPA-positive patients with RA. Their experimental data suggested that IgM-RF enhanced the capacity of ACPA immune complexes to further stimulate cytokine production by macrophages, and consequently that RF would affect the immune process and/or the pathogenicity of ACPA immune complexes in RA [15, 16]. These results increased interest in the timing of the appearance of the different antibodies during the pre-disease period.

The aim of this study was to investigate the interplay between three isotypes of RF with ten different ACPA fine specificities, anti-CCP2 and anti-CarP antibodies analysed previously in samples collected from individuals before symptom onset and following a diagnosis of RA in comparison with population control subjects.

## Methods

### Subjects

A case–control study was conducted with individuals included in population surveys within the Medical Biobank of Northern Sweden and the Maternity cohort. The criteria for recruitment, and the collection and storage of blood samples, have been described in detail previously [7]. Cohorts included in the Medical Biobank are population-based, and all adult individuals residing in the county of Västerbotten are continuously invited to participate. The Maternity cohort is a collection of serum samples obtained from pregnant women in northern Sweden undergoing screening for immunity to rubella [7]. The registers from the Medical Biobank and the Maternity cohort were co-analysed with the registers of patients fulfilling the 1987 American Rheumatism Association classification criteria for RA [17] and attending the Department of Rheumatology, University Hospital, in Umeå, Sweden, with a known date for their awareness of symptoms to identify individuals having donated blood samples before the onset of symptoms of RA. The number of individuals identified and the procedure for excluding any sample are described in detail elsewhere [9]. Consequently, 386 individuals, referred to as *pre-symptomatic individuals*, having donated a total of 717 blood samples (476 from the Medical Biobank and 241 from the Maternity cohort) at different time points were included in this study. Before 1988, samples from the Maternity cohort were routinely heat-inactivated; therefore, such samples from pre-symptomatic individuals were excluded from further analysis of RF, leaving 598 samples from 321 pre-symptomatic individuals. Of these 321 individuals, 171 contributed with 1 sample, 75 with 2 samples, 42 with 3 samples, 20 with 4 samples, 7 with 5 samples and 6 with 6 samples. The median [interquartile range (IQR)] time pre-dating the onset of symptoms using all 598 samples was 6.2 (7.2) years. Samples in the Maternity cohort were generally obtained earlier; the median (IQR) pre-dating time was 8.7 (9.5) years, and for samples from the Medical Biobank of Northern Sweden the predating time was 5.6 (6.9) years. Control subjects (*n* = 1305) were randomly identified from the same cohorts within the registers of the Medical Biobank of Northern Sweden and the Maternity cohort, respectively, and matched for age, sex and date of blood sampling. For analysis of RF isotypes, 492 individuals were randomly selected from among the 1305 control subjects. Of the 321 pre-symptomatic individuals, 187 were also sampled when they presented at the early arthritis clinic (Department of Rheumatology) and diagnosed with RA, representing a cohort of patients with early RA. Thus, these patients with early RA had samples collected before (included in the pre-symptomatic group) and after onset of symptoms (included in the early RA group). The median (IQR) duration before these patients were diagnosed following the onset of symptoms was 7.4 (5.9) months. Human leucocyte antigen (HLA) typing was performed as previously described [18]. Demographic data of pre-symptomatic individuals, patients with RA and control subjects are presented in Table 1. The regional ethics committee at the University Hospital, Umeå, Sweden, approved this study, and all participants gave their written informed consent when donating blood samples.

**Table 1:** Demographic data for the 321 pre-symptomatic, 187 patients with RA and 492 control subjects

	Control subjects (*n* = 492)	Pre-symptomatic individuals (*n* = 321)	RA patients (*n* = 187)
Female sex, %	71.5	77.9	74.3
Median age, yr (IQR)	51.1 (20.1)	50.3 (20.0)^a^	57.1 (15.3)
Ever smoker, *n*/total (%)	221/462 (47.8)	197/308 (64.0)^b^	123/184 (66.8)^b^
HLA-shared epitope^c^, *n* (%)	224/481 (46.5)	198/304 (65.1)^b^	118/187 (63.1)^b^

*RA* rheumatoid arthritis, *IQR* interquartile range, *HLA* human leucocyte antigen

^a^Median age, as calculated for all samples when collected (*n* = 598) at a median (IQR) of 62. (7.2) years before the onset of RA symptoms

^b^ *p* < 0.001 cases compared with controls

^c^HLA-shared epitope = HLA-B1*0101/0401/0404/0405/0408

### Rheumatoid factor analysis

Analysis of RF of the IgA, IgG and IgM isotypes was performed using the EliA immunoassay on the Phadia 2500 system according to the manufacturer’s instructions (Phadia GmbH, Freiburg, Germany). Cut-off values for the RF isotypes were decided using receiver operating characteristic (ROC) curves with the optimum of sensitivity and specificity, which in all three cases yielded 96 % specificity.

### Multiplex assay

Serum and/or plasma samples were analysed for levels of ACPA specificities of the IgG isotype using a custom-made microarray based on the ImmunoCAP ISAC system (Phadia AB, Uppsala, Sweden) as previously described [19]. Briefly, ten different citrullinated antigens were analysed as previously presented [9]: Fibα_563–583_ (citrullinated at position 573), Fibα_580–600_ (citrullinated at position 591), Fibβ_62–81a_ (citrullinated at position 72), Fibβ_62–81b_ (citrullinated at position 74), Fibβ_36–52_ (citrullinated at position 44), α-enolase (CEP-1/Eno_5–21_) (citrullinated at positions 10 and 16), triple helical collagen type II (citC1 CII_359–369_ with citrulline at positions 360 and 365), filaggrin (CCP-1/Fil_307–324_), vimentin (Vim)_2–17_ (citrullinated at positions 4, 12 and 13) and Vim_60–75_ (citrullinated at position 64, 69 and 71). For all reactivities, both the citrullinated and native arginine-containing peptides were analysed, and for all except one, citC1, the determined Δ values were used. The exception was due to the arginine containing citC1 being inherently an autoantigen [20]. The cut-off values were based on ROC curves and have been presented previously [9].

### Analysis of anti-CarP and anti-CCP2 antibodies

The anti-CarP antibodies were determined using carbamylated (Car) foetal calf serum (FCS) as the detection antigen, as described previously [10, 12]. Briefly, MaxiSorp plates (Nunc, Roskilde, Denmark) were coated with 10 μg/ml FCS (Bodinco, Alkmaar, the Netherlands) and Car-FCS at 4 °C overnight. The plates were blocked with 1 % bovine serum albumin (Sigma-Aldrich, St. Louis, MO, USA) at 4 °C for 6 h, followed by incubation with 1:50 diluted sera on ice overnight. Bound antibodies were detected with rabbit anti-human IgG horseradish peroxidase (Dako, Glostrup, Denmark) on ice for 4 h and subsequently visualized with 2,2-azino-bis(3-ethylbenzothiazoline-6-sulfonate). Absorbance was measured at 415 nm and transformed to arbitrary units per millilitre (AU/ml) using a titration curve of a serum pool from three anti-CarP–antibody positive samples. The background signal of FCS was subtracted from the signal of Car-FCS to analyse the specific anti-CarP antibody reactivity. The cut-off for positivity was set at 256.1 AU/ml, giving a specificity of 97 %. Samples analysed for anti-CarP, including those collected <13.0 years before onset of symptoms and taking into account those lost due to lack of samples (*n* = 106 for pre-symptomatic individuals), a subset of control subjects with similar sex and age distribution was selected, resulting in 421 samples analysed from 252 pre-symptomatic individuals, 150 from control subjects and 181 from patients with RA. The cut-off level for anti-CarP antibodies was defined using ROC curves as previous described [12]. The cut-off for positivity was set at 256.07 AU/ml, giving a specificity of 97 %. Antibodies against CCP2 were analysed using enzyme-linked immunosorbent assay according to the manufacturer’s instructions (Euro Diagnostica, Malmö, Sweden), with a cut-off for positivity set at 25 AU/ml according to the manufacturer’s instructions.

### Statistics

For comparisons of continuous data between two groups, the Mann-Whitney *U* test was used. The Kruskal-Wallis test was used for several groups. Correlation analyses were performed using Spearman’s rank correlation test (*r*_s_). Univariate analyses of variance were used to identify associations between factors and continuous data. Relationships between categorical data (positive vs. negative) were compared using χ^2^ analysis or Fisher’s exact test as appropriate. Considering the study to be explorative, *p* values ≤0.05 were considered significant. Logistic regression analyses were performed to identify associations between antibodies and disease development, presented as odds ratios (ORs) and 95 % confidence intervals (95 % CI). In the logistic regression analysis, the highest OR is achieved when controlled for other variables that are present in the model. Instead of an array of logistic regressions, conditional inference tree modelling was used. Conditional inference tree analysis identifies first the explanatory variable (EV; i.e., antibody) having the strongest association with the response variable (pre-symptomatic vs. control individuals) and splits the data based on the cut-off value of the selected EV [21]. This results in two branches where each branch contains all subjects whose observed value for the selected EV is below or above the cut-off, respectively. In the next step, the EV that has the strongest association among the remaining EVs is selected for each branch separately, and this process is repeated. A branch will not be split if there are no EVs with a strong enough association with the response variable. When the splitting of branches has stopped, the relative frequencies of cases to controls at the endpoints (the leaves) are used as estimated probabilities of a randomly selected subject’s belonging to the particular leaf being a case or control; this probability is estimated conditional on the EVs and their cut-off values along the path leading to the leaf. Statistical calculations were performed using IBM SPSS for Windows version 22 software (IBM, Armonk, NY, USA) and R software (R Core Team, 2014) [22]. Sensitivity, specificity, OR and 95 % CI were calculated with the XLSTAT program (version 2014.1.04) in Microsoft Excel 2013 (Addinsoft). Euler diagrams were created using the eulerAPE 3.0.0 freeware [23].

## Results

### Levels of RF isotypes

The concentrations of the three RF isotypes differed significantly between the pre-symptomatic individuals and control subjects, respectively, presented as median (IQR): IgA-RF 3.2 (4.2) IU/ml vs. 1.3 (2) IU/ml, IgG-RF 5.0 (5) μg/L vs. 3.0 (2) μg/L and IgM-RF 2.0 (9.3) IU/ml vs. 1.0 (1) IU/ml (*p* < 0.001 for all). The concentrations after the onset of disease [median (IQR) IgA-RF 11.0 (23) IU/ml, IgG-RF 12.0 (22) μg/L, and IgM-RF 44.0 (109) IU/ml] were significantly increased compared with those for both the control subjects and pre-symptomatic individuals (*p* < 0.0001 by Kruskal-Wallis test). The concentration of IgM-RF increased significantly during the pre-dating time (*p* < 0.001) and on an individual level (*p* < 0.0001 by Friedman’s test; data not shown).

### Frequencies of RF isotypes

The frequency of RF positivity of the IgA, IgG and IgM isotypes in all samples from the pre-symptomatic individuals was 25 %, 18 % and 26 %, respectively (Table 2), and from patients at the time of diagnosis it was 64 %, 57 % and 79 %, respectively (data not shown). The frequencies and OR (95 % CI) for all ten of the ACPA, anti-CCP2 and anti-CarP antibodies analysed are presented in Table 2 for comparison.

**Table 2:** Diagnostic accuracy of ten ACPAs (presented in alphabetical order), anti-CCP2 antibodies, anti-CarP antibodies and RF isotypes, analysed separately and in combination with the three RF isotypes, for RA development in pre-symptomatic individuals

Antibody	Sensitivity (95 % CI)	Odds ratio (95 % CI)	+ IgA-RF OR (95 % CI)^a^	+ IgG-RF OR (95 % CI)^a^	+ IgM-RF OR (95 % CI)^a^
CCP-2	29.6 (26.08–33.39)	20.26 (10.58–38.83)	50.7 (12.4–207.5)	30.2 (7.3–124.9)	67.6 (16.6–275.9)
CEP-1	23.9 (20.64–27.51)	4.75 (3.14–7.2)	19.6 (7.1–54.4)	13.3 (4.7–37.3)	48.1 (11.7–197.4)
CitC1^III^	12.71 (10.26–15.66)	3.77 (2.22–6.4)	36.9 (5.0–271.5)^b^	25.1 (3.4–186.9)	45.7 (6.3–333.6)
Fibα_563–583_	7.63 (5.74–10.08)	1.65 (0.99–2.78)	21.7 (2.9–162)	11.8 (1.5–91.7)	13.9 (3.3–59.2)
Fibα_580–600_	6.95 (5.16–9.32)	1.05 (0.65–1.7)	6.2 (1.8–21.5)	6.3 (1.8–21.7)	5.2 (1.5–18.3)
Fibβ_36–52_	22.54 (19.36–26.1)	3.98 (2.66–5.95)	17.5 (6.3–48.6)	15 (4.6–49)	45.2 (11–185.7)
Fibβ_62–81a_	8.64 (6.63–11.22)	2.77 (1.56–4.92)	17.5 (2.3–131.4)	9.1 (1.1–72.8)	18.1 (2.4–135.8)
Fibβ_62–81b_	13.39 (10.88–16.4)	9.2 (4.4–19.24)	42.6 (5.8–311.7)^b^	24.8 (3.3–183.9)^b^	50.2 (6.9–366.2)^b^
Filaggrin	21.86 (18.72–25.39)	9.39 (5.33–16.55)	22.9 (7.1–73.6)	48.4 (6.6–353.4)	37.9 (9.2–156)
Vim_2–17_	5.59 (4–7.79)	1.31 (0.75–2.29)	7.6 (1.7–33.5)	10.1 (1.3–79.6)	13.6 (1.8–104.3)
Vim_60–75_	9.49 (7.38–12.15)	1.93 (1.18–3.14)	8.1 (2.4–27.3)	9.1 (2.1–39.5)	9.3 (2.8–30.8)
CarP	13.78 (10.81–17.43)	5.83 (2.08–16.36)	12.9 (1.7–95.7)^b^	10.6 (1.4–78.6)	16.2 (2.2–119.2)^b^
IgA-RF	24.75 (21.46–28.37)	9.07 (5.5–14.96)^a^	–	23.9 (7.4–76.7)	21.9 (8.8–54.6)
IgG-RF	17.56 (14.72–20.83)	5.89 (3.53–9.82)^a^	23.9 (7.4–76.7)	–	34.5 (8.4–142.2)
IgM-RF	26.09 (22.73–29.76)	11.08 (6.64–18.5)^a^	21.9 (8.8–54.6)	34.5 (8.4–142.2)	–

*ACPA* anti-citrullinated protein antibodies, *anti-CarP* antibodies against carbamylated proteins, *anti-CCP* anti-cyclic citrullinated peptide antibodies, *CEP-1* α-enolase, *CI* confidence interval, *Fib* fibrinogen, *IgA* immunoglobulin A, *IgG* immunoglobulin G, *IgM* immunoglobulin M, *RF* rheumatoid factor, *Vim* vimentin

Data represent pre-symptomatic individuals with 598 samples. Logistic regression analysis was performed with the combinations using two groups (++ vs. all other combinations).

^a^Calculation adjusted for age at the time of sampling

^b^Calculations made with a hypothetical control individual double positive for the respective antibodies

To exclude the possibility that pregnancy as such could have any impact on the findings, the control subjects were selected randomly from among the same cohorts as the pre-symptomatic individuals; that is, the pre-symptomatic individuals from the Medical Biobank of Northern Sweden were assigned control subjects from that cohort (population survey project), and the pre-symptomatic individuals from the Maternity cohort were assigned to control subjects from the same cohort.

### Relationships between RF isotypes and ACPA, anti-CCP2 and/or anti-CarP antibodies

**Fig. 1:**
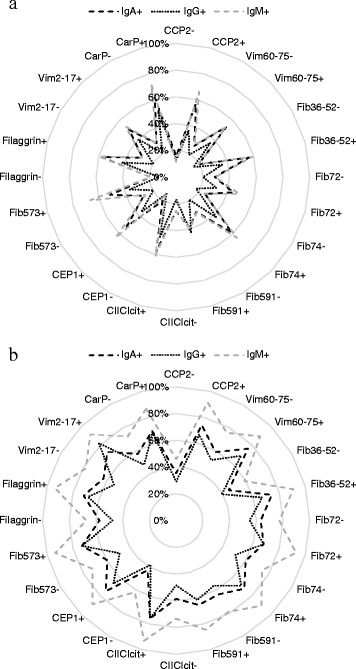
Polar chart depicting the frequencies for ever being positive for the combinations of the ten ACPA and anti-CCP2 and anti-CarP antibodies with IgA-, IgG- and IgM-RF isotypes in pre-symptomatic individuals (**a**) and patients with RA (**b**). *CCP2* anti-CCP2 antibodies, *Vim60-75* anti-vimentin 60-75 antibodies, *Fib36-52* anti-fibrinogen (Fib)36-52 antibodies, *Fib72* anti-Fibβ62-81a antibodies, *Fib74* anti-Fibβ62-81b antibodies, *Fib591* anti-Fibα580-600 antibodies, *CIIC1cit* anti-citC1 CII 359-369 antibodies, *CEP1* anti-α-enolase antibodies, *Fib573* anti-Fibα563-583 antibodies, *filaggrin* anti-filaggrin antibodies, *Vim2-17* anti-vimentin2-17 antibodies, *CarP* anti-CarP antibodies, *RA* rheumatoid arthritis, + = positivity, − = negativity

The frequency of ever being positive for each RF isotype among samples negative for the different ACPA in the pre-symptomatic individuals were as follows: for IgA-RF, 19–30 %; for IgG-RF, 13–20 %; and for IgM-RF, 16–31 %. The frequency of positivity among the anti-CCP2− individuals was generally slightly lower (16 %, 13 % and 12 %, respectively), and in anti-CarP− samples it was slightly higher (29 %, 21 % and 29 %, respectively) (Fig. 1a). The highest frequency in the pre-symptomatic individuals for combinations with the different RF isotypes was found for anti-CarP antibodies, showing a frequency of 71 % for IgM-RF, followed by 62 % and 51 % for the IgA and IgG isotypes, respectively (Fig. 1a). At the time of diagnosis, the frequencies of positivity for RF isotypes in ACPA− individuals were greatly increased compared with pre-symptomatic individuals (Fig. 1b). The concentrations of all three RF isotypes were found to correlate with an increasing number of ACPA in pre-symptomatic individuals (*r*_s_ = 0.328–0.338, *p* < 0.001).

**Fig. 2:**
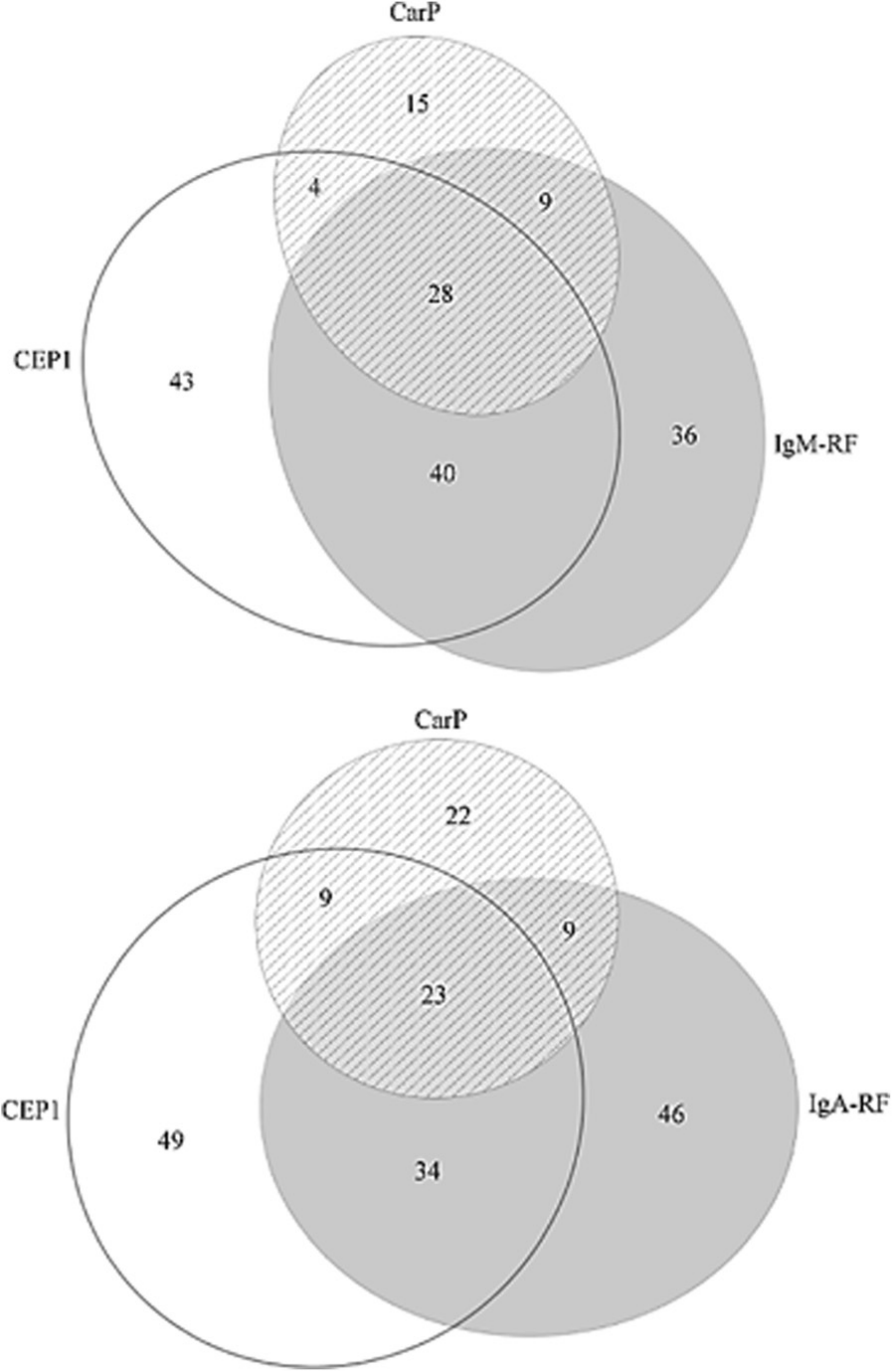
Proportional Euler diagrams illustrating the relationships between, as an example, one anti-citrullinated protein antibody (ACPA), anti-CEP1 antibody (CEP1), immunoglobulin M rheumatoid factor (IgM-RF)/IgA-RF and anti-carbamylated protein antibodies (CarP). The numbers indicate the number of individuals positive for the antibody/RF of the pre-symptomatic individuals for each factor or combinations

In samples from pre-symptomatic individuals, the concentrations of IgA-RF and IgM-RF correlated with the concentrations of all ten ACPA specificities analysed (*r*_s_ = 0.09–0.27, *p* < 0.001–0.05) except for Vim_2–17_, whilst IgG-RF correlated with all ACPA (*r*_s_ range 0.1–0.28, *p* < 0.001–0.05) except for Fibα_563–583_ and Fibβ_62–81a_. The concentration of anti-CarP antibodies and anti-CCP2 correlated with IgA-, IgG- and IgM-RF isotypes present in samples from pre-symptomatic individuals (for anti-CarP antibodies, *r*_s_ = 0.24, 0.22 and 0.29, respectively; for anti-CCP2 antibodies, *r*_s_ = 0.24, 0.22 and 0.52, respectively; *p* < 0.0001 for all). The relationships between ACPA, anti-CarP antibodies and RF isotypes (IgM and IgA) are presented in Euler diagrams, including one of the most frequently appearing ACPA (anti-CEP1 antibodies), anti-CarP antibodies and IgM-RF or IgA-RF (Fig. 2). For the other ACPA of similar frequencies (anti-Fib36-52 and anti-filaggrin antibodies), the diagrams were similar; for those of lower frequencies, there was less overlap.

The OR for development of RA was increased greatly by the combinations of seropositivity for any of the three RF isotypes and any of the ACPA in the pre-symptomatic individuals (Table 2). There were no control individuals positive for the combination of anti-CarP antibodies and IgA-RF or IgM-RF; however, in calculations using a hypothetical double-positive sample for one randomly selected control, the ORs of these combinations also yielded a high OR for disease development (Table 2). The frequency of double-positivity of ACPA and RF of any isotype in the same sample increased significantly over time with the increase detected the closer to the onset of symptoms (time stratified into 5-year groups) compared with being double-negative or positive for only one of the antibodies (χ^2^ = 31.91, 9 *df*, *p* < 0.001).

Combinations of anti-CCP2 antibodies and seropositivity for one, two or three of the analysed RF isotypes yielded high ORs (39.5–44.8) without an increase in OR regardless of the number of RF isotypes (Table 3). A similar pattern was observed for anti-filaggrin antibodies in combination with any number of RF isotypes, although with slightly lower ORs (25.3–29.5). For anti-CEP-1 and anti-Fibβ_36–52_ antibodies, the combinations with the number of RFs showed an increasing OR with an increasing number of RF isotypes (15.5–33.2 and 14.5–30.5, respectively); however, analysis of combinations of the triplet (anti-CEP-1, anti-Fibβ_36–52_ and anti-filaggrin antibodies) yielded decreasing ORs (32.3–16.9) with overlapping CIs. It was not possible to compute combinations with anti-CarP antibodies, owing to the lack of control subjects positive for the combinations (Table 3). However, the highest specificity (100 %) was found for anti-CarP antibodies in combination with IgA-RF or IgM-RF, anti-CCP2 antibodies or ACPA (anti-Fibβ_36–52_, anti-CEP-1 and anti-filaggrin antibodies) (Table 3). A comparison within pre-symptomatic individuals and control subjects of the combinations of the ten different ACPAs and/or seropositivity for having one or more, or two or more, of the three RF isotypes and/or presence of anti-CarP antibodies yielded in general a higher sensitivity with a small decrease in specificity compared with having ACPA alone (Additional file 1: Table S1.).

**Table 3:** Prevalence, sensitivity, specificity and OR of numbers of RF isotypes, alone and in combination with the three most frequent ACPA specificities, anti-CCP2 antibodies and anti-carbamylated protein antibodies in pre-symptomatic individuals and population control subjects

Antibody/combination	Pre-symptomatic (*n*)	Control subjects (*n*)	Sensitivity % (95 % CI)	Specificity % (95 % CI)	OR (95 % CI)
1 RF	123	39	20.6 (17.5–24)	92.1 (89.3–94.2)	3 (2.1–4.4)
2 RF	56	7	9.4 (7.3–12)	98.6 (97–99.4)	7.2 (3.2–15.9)
3 RF	58	1	9.7 (7.6–12.4)	99.8 (98.7–100)	52.7 (7.3–382.2)
≥1 RF	237	47	39.6 (35.8–43.6)	90.4 (87.5–92.7)	6.2 (4.4–8.8)
≥2 RF	114	8	19.1 (16.1–22.4)	98.4 (96.7–99.2)	14.2 (6.9–29.5)
3 RF	58	1	9.7 (7.6–12.4)	99.8 (98.7–100)	52.7 (7.3–382.2)
CCP2 + ≥1 RF	124	3	20.7 (17.7–24.2)	99.4 (98.1–99.9)	42.6 (13.5–135)
CCP2+ ≥2 RF	83	2	13.9 (11.3–16.9)	99.6 (98.4–100)	39.5 (9.7–161.4)
CCP2 + 3 RF	50	1	8.4 (6.4–10.9)	99.8 (98.7–100)	44.8 (6.2–325.5)
CCP2 + ≥1 RF + CarP	35	0	6.1 (4.4–8.4)	100 (99–100)	–
CCP2+ ≥2 RF+ CarP	30	0	5.1 (3.6–7.3)	100 (99–100)	–
CCP2 + 3 RF+ CarP	22	0	3.7 (2.5–5.6)	100 (99–100)	–
CEP-1 + ≥1 RF	96	6	16.3 (13.5–19.5)	98.8 (97.2–99.5)	15.5 (6.7–35.7)
CEP-1+ ≥2 RF	64	3	10.8 (8.6–13.6)	99.4 (98.1–99.9)	19.5 (6.1–62.5)
CEP-1+ 3 RF	38	1	6.4 (4.7–8.7)	99.8 (98.7–100)	33.2 (4.5–243.1)
CEP-1 + ≥1 RF + CarP	28	0	4.9 (3.4–7)	100 (99–100)	–
CEP-1+ ≥2 RF + CarP	25	0	4.3 (2.9–6.3)	100 (99–100)	–
CEP-1+ 3 RF + CarP	17	0	2.9 (1.8–4.7)	100 (99–100)	–
Fibβ_36–52_ + ≥1 RF	91	6	15.4 (12.7–18.6)	98.8 (97.2–99.5)	14.5 (6.3–33.5)
Fibβ_36–52_ + ≥2 RF	57	2	9.7 (7.5–12.3)	99.6 (98.4–100)	25.8 (6.3–106.1)
Fibβ_36–52_ + 3 RF	35	1	5.9 (4.3–8.2)	99.8 (98.7–100)	30.5 (4.2–223.2)
Fibβ_36–52_ + ≥1 RF + CarP	23	0	4 (2.7–6)	100 (99–100)	–
Fibβ_36–52_ + ≥2 RF + CarP	20	0	3.4 (2.2–5.3)	100 (99–100)	–
Fibβ_36–52_ + 3 RF + CarP	14	0	2.4 (1.4–4)	100 (99–100)	–
Filaggrin + ≥1 RF	85	3	14.4 (11.8–17.5)	99.4 (98.1–99.9)	27 (8.5–85.9)
Filaggrin + ≥2 RF	56	2	9.5 (7.4–12.2)	99.6 (98.4–100)	25.3 (6.1–104.1)
Filaggrin + 3 RF	34	1	5.8 (4.1–8)	99.8 (98.7–100)	29.5 (4–216.6)
Filaggrin + ≥1 RF + CarP	23	0	4 (2.7–6)	100 (99–100)	–
Filaggrin + ≥2 RF+ CarP	20	0	3.4 (2.2–5.3)	100 (99–100)	–
Filaggrin + 3 RF + CarP	14	0	2.4 (1.4–4)	100 (99–100)	–
CEP-1 + Fibβ_36–52_ + Filaggrin + ≥1 RF	37	1	6.3 (4.6–8.6)	99.8 (98.7–100)	32.3 (4.4–236.4)
CEP-1 + Fibβ_36–52_ + Filaggrin + ≥2 RF	28	1	4.7 (3.3–6.8)	99.8 (98.7–100)	24.1 (3.3–177.5)
CEP-1 + Fibβ_36–52_ + Filaggrin + 3 RF	20	1	3.4 (2.2–5.2)	99.8 (98.7–100)	16.9 (2.3–126.7)
CEP-1 + Fibβ_36–52_ + Filaggrin + ≥1 RF + CarP	13	0	2.2 (1.3–3.8)	100 (99–100)	–
CEP-1 + Fibβ_36–52_ + Filaggrin + ≥2 RF + CarP	12	0	2 (1.1–3.6)	100 (99–100)	–
CEP-1 + Fibβ_36–52_ + Filaggrin + 3 RF + CarP	10	0	1.7 (0.9–3.2)	100 (99–100)	–

*RF* rheumatoid factor, *CCP2* anti-CCP2 antibodies, *Fibβ*_*36–52*_ Fibrinogenβ_36–52_, *CEP-1* anti-α-enolase antibodies

### Conditional inference tree analysis including all antibodies

Conditional inference tree analysis was performed to split the subjects into groups of differing probabilities of future disease development. For the analysis, we included samples from control subjects (*n* = 472) and pre-symptomatic individuals (*n* = 122) collected within the 3 years closest to symptom onset, with only one sample per individual, using the dichotomised variable of the ten analysed ACPA, three RF isotypes and anti-CCP2, age at time of sampling (older or younger than 50 years), having HLA–shared epitope (HLA-SE) or not, and ever being a smoker or not. Of the included variables, anti-CCP2 antibody positivity provided the most discriminative starting split (Additional file 2: Figure S1). For subjects positive for anti-CCP2, anti-filaggrin was the next antibody to be detected (*p* < 0.001). Positivity for both of these antibodies identified cases from the control subjects with a probability of 97 %. The second highest probability to identify pre-symptomatic individuals was in the anti-CCP2+, anti-filaggrin− and anti-CEP-1+ group (94 % probability). The overall effect of this model yielded a sensitivity of 53.7 %, a specificity of 97.6 %, a positive predictive value (PPV) of 85.7 % and a negative predictive value (NPV) of 88.6 % for disease development ≤3 years before onset of symptoms. Testing several different models with and without smoking or age and including only the antibodies of highest frequency (i.e., anti-CEP-1, anti-Fibβ36-52, anti-filaggrin, anti-CCP2 antibodies and all three RF isotypes) did not change the model presented in Additional file 2: Figure S1. Removing HLA-SE from the model affected only the HLA-SE branch, to be changed to anti-Fibβ62-81a antibodies. Because filaggrin and CCP2 peptides share the same background, a model not including anti-filaggrin antibodies but otherwise identical to model 1 was tested (Additional file 2: Figure S1). This resulted in a model identical to model 1 but with anti-filaggrin antibodies exchanged for IgM-RF (i.e., IgM-RF being the second most discriminative variable for both anti-CCP2+ and anti-CCP2− individuals).

Because the anti-CarP antibody was analysed in fewer of the control and pre-symptomatic samples, we included the anti-CarP antibody results in a separate model (*n* = 143 and *n* = 93, respectively). That model included all variables used to construct model 1 and the dichotomised value of anti-CarP antibodies resulting in anti-CCP2 antibody test still being the most discriminative variable. The second antibody detectable if individuals were positive for anti-CCP2 antibodies was IgM-RF, and positivity for both antibodies yielded 100 % probability of detecting pre-symptomatic individuals who would develop RA within 3 years, with a sensitivity of 68.3 %, a specificity if 93.3 %, PPV of 87.7 % and NPV of 80.9 %.

### Timing of rheumatoid factor and ACPA in pre-symptomatic individuals

**Fig. 3:**
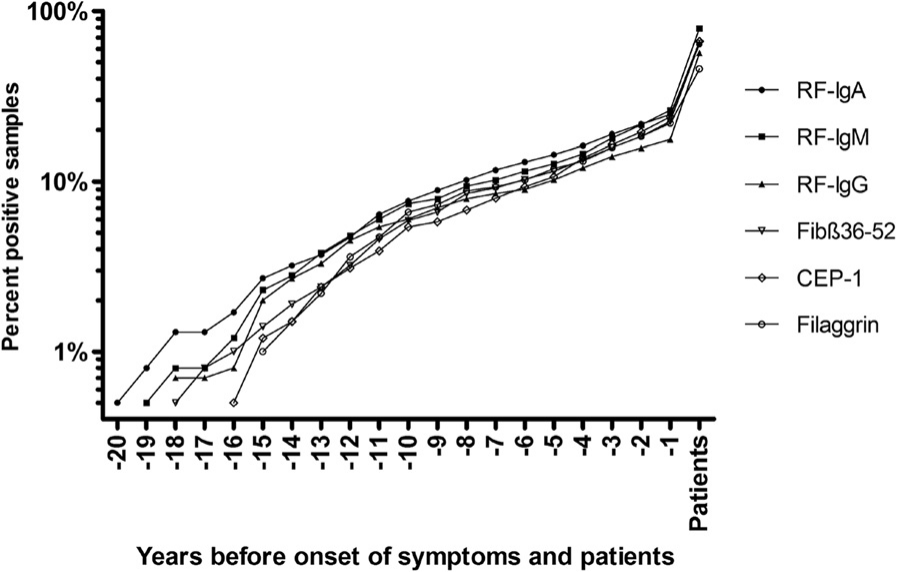
Cumulative percentage of positivity for rheumatoid factor of immunoglobulin A (IgA), IgG and IgM isotypes and antibodies against fibrinogen (Fib)β_36–52_, α-enolase (CEP-1), filaggrin and carbamylated protein (CarP) including all pre-symptomatic samples

The frequency of positivity during the whole pre-dating time of the RF isotypes increased significantly only for IgM-RF in analysis of samples before onset of symptoms (*p* < 0.05 by χ^2^ trend test) and for the ACPA, anti-Fibβ_36–52_, anti-CEP-1, anti-Fibα_563–583_ and anti-Vim_2–17_ antibodies (*p* < 0.01–0.05 by χ^2^ trend test) and anti-CCP2 antibodies (*p* < 0.001 by χ^2^ trend test). The most frequently appearing antibodies are presented in Fig. 3.

The RF isotypes were the antibodies appearing first when compared with ACPA and anti-CCP2 antibodies. In samples collected ≥15 years before the onset of symptoms, 26.3 % were IgA-RF+, 18.4 % IgM-RF+ and 13.2 % IgG-RF+, whilst the most frequently appearing ACPA was anti-Fibβ_36–52_, with 15.8 % positivity (illustrated in Fig. 3). These frequencies were significantly higher for IgA-RF (*p* < 0.05) and trend for significant for IgM-RF (*p* = 0.063). However, there were no significant differences between the various combinations of antibodies. Analyses at the individual antibody level did not confirm any differences, because the primary antibody appearing was either an RF isotype or ACPA (evaluated as positivity for anti-CEP-1, anti-Fibβ_36–52_ or anti-Fib_307–324_ antibodies) but not anti-CCP2.

The OR for disease development when positive for all three RF isotypes was very high at 83 (95 % CI 11.3–611.3) in pre-symptomatic individuals whose samples were collected less than 5 years before onset of symptoms, with only a minor variation in OR for time groups closer to disease onset, ranging from 92.1 to 101.8 (data not shown).

Positivity for IgM-RF or IgA-RF in combination with ACPA specificities anti-CEP-1, anti-Fibβ_36–52_, anti-Fibα_580–600_, anti-filaggrin antibodies and anti-CCP2 antibodies was associated with a significantly shorter time to onset of symptoms (*p* < 0.001–0.05). Also, the ORs were higher (range 13.7–71.1) for these combinations compared with being negative for both or positive for only one (data not shown). Applying the same analyses for these combinations to samples acquired <5 years before onset of symptoms yielded even higher ORs (range 15.6–101.4).

## Discussion

In this study, the presence of RFs of IgA, IgG and IgM isotypes was evaluated and related to the simultaneous presence of anti-CCP2 and anti-CarP antibodies and ten different ACPA fine specificities using samples from pre-symptomatic individuals, population control subjects and patients with RA. Cut-off levels for positivity were determined using ROC curves for the RFs in addition to, and as previously performed for, ACPA specificities and anti-CarP antibodies [9, 12]. The specificity of all the antibodies analysed was between 95 % and 98 %. The highest sensitivity in the pre-symptomatic individuals was for anti-CCP2 (29.6 %), followed by IgM-RF (26.1 %), IgA-RF (24.8 %) and CEP-1 (23.9 %) when we analysed all samples from the pre-symptomatic individuals with a median pre-dating time of 6.2 years before the onset of symptoms.

In order to determine whether there was a time difference for the antibodies appearing first, RF and the analysed ACPA and anti-CCP2 antibodies were compared in samples collected ≥15 years before onset of symptoms. Seropositivity for IgA-RF was significantly more frequent in this time period compared with the ACPA most frequently detectable (i.e., anti-Fibβ_36–52_) and borderline significant for IgM-RF (*p* = 0.06). In a previous study, we showed that IgA-RF occurred in higher frequencies, and to be a better predictor for disease development, than IgM-RF during the early pre-symptomatic period [5, 7]. Furthermore, we previously reported that IgM-RF increases in frequency the closer to symptom onset, similar to the results presented here [7]. Interestingly, in two recent publications, researchers have presented data suggesting that the presence of IgM-RF augmented macrophage stimulation in vitro by ACPA immune complexes compared with ACPA alone [15, 16]. Those results suggest a mechanistic link between IgM-RF and ACPA, and a contribution of this interaction to the pathogenesis of RA. An earlier presence of RF(s) would, in that case, be an enhancer for disease development. In this study, the highest OR for development of RA was the combination of positivity for anti-CCP2 antibodies and IgM-RF, yielding an OR of 67.6 using all other combinations as a reference (double-negative or only one of the antibodies positive) (Table 2). In general, combinations of IgM-RF and ACPA specificities yielded the highest ORs, with the exception of antibodies against filaggrin and IgG-RF and against Fibα_563–583_ in combination with IgA-RF.

By using a conditional inference tree analysis, it was possible to identify those antibodies contributing to the highest probability for disease development ≤3 years pre-dating symptom onset when all antibodies (except anti-CarP antibodies, due to the reduced sample size) were taken into account as well as HLA-SE, smoking habit and age at the time of sampling. By this analysis, it was found that anti-CCP2 positivity provided the best separation of pre-symptomatic individuals sampled <3 years before onset of symptoms from control subjects, followed by positivity for anti-filaggrin antibodies, yielded a probability of detecting a pre-symptomatic individuals of 97 %. In the same model, the second highest probability was found for anti-CCP2 positivity, anti-filaggrin antibody negativity and anti-CEP-1 antibody positivity with 94 % probability of detecting an individual who would develop RA within 3 years. Using this model, the overall specificity was found to be relatively high, 97.5 %, with a modest sensitivity of 54 % and a PPV of 85.7 %. This modest sensitivity overall is probably a reflection of the poor abilities of the other factors and antibodies in the model to define cases on the left-hand side of the model (starting with anti-CCP2 negativity) (Additional file 2: Figure S1). Despite this, we consider that this model, added to the current predictive information available for individuals who will develop RA later, especially these combinations of antibodies will provide a high probability of later developing RA. However, when we added anti-CarP antibodies to the model, IgM-RF became the second antibody to predict disease development, and the sensitivity for the model increased to almost 70 % with a good specificity of 93 %. This supports a contribution of anti-CarP antibodies being related to IgM-RF for identifying individuals who will develop RA.

The frequencies of RF isotypes calculated for the pre-symptomatic individuals in the present study are somewhat different from the results presented by Gan and co-workers [13]. This is considered to be due, at least partly, to the different methods used for determining the cut-off limits. The results presented by Gan et al. [13], when they applied the <5 % definition as the cut-off level RFs of IgM and IgA isotypes, and all combinations [CCP2 positivity combined with the number of RFs (≥1 RF or ≥2 RF) and/or CarP-antibodies] showed a higher sensitivity and a lower specificity compared with the results of this study, with the exception of anti-CCP2 antibodies and IgG-RF. When they applied the ≥2 SD cut-off, the results were similar, except for IgG-RF having a lower specificity. Nonetheless, the prevalence of the three isotypes in pre-symptomatic individuals were in the same order in both studies, with IgM-RF displaying the highest frequency, followed tightly by IgA-RF and with IgG-RF having a distinctly lower frequency. In this comparison between the autoantibodies and RFs, all samples were included, in contrast to Gan et al., who used only ever-positive samples for their calculations, with a consequent increase in sensitivity [13]. It is currently not evident how the different antibody reactivities (e.g., ACPA, anti-CarP or RFs) contribute to the pathogenesis of RA. We have proposed also in support of other studies [15, 16] a mechanistic link between IgM-RF and ACPA. However, there is insufficient data in humans or experimental models to explain the development and interplay between these different antibodies.

Although it was possible to analyse samples from one of the largest patient cohorts available, with samples identified sequentially before the ascertained date for the onset of disease symptoms, identification of any relationships between the different antibodies and statistical calculations remained limited. The samples were not, at the individual level, collected at regular intervals, and by stratifying the data the number of points for statistical calculation was reduced. About 20 % of the samples for both cases and control subjects were collected within the Maternity cohort (i.e., from pregnant women). Antibody frequencies in the pre-symptomatic individuals from this cohort were lower than among samples from the Medical Biobank as a result of generally longer storing pre-dating time for the Maternity cohort. Thus, our results did not indicate that pregnancy per se would increase the ACPA frequencies in pre-symptomatic individuals. The number of data points was also reduced, as the treatment of samples before 1988 affected the analyses of RFs, and the age of the individuals also affected the concentrations of RFs, which was also taken into account. Another matter of concern is the different sensitivities of the assays for detecting the various antibodies possibly resulting in different specificity of the tests.

## Conclusions

On the basis of these data, we conclude that IgM-RF provides a good prognostic valuation, especially in combination with certain ACPA fine specificities, for disease development in samples collected before the onset of symptoms of RA. Our results provide an increased knowledge of the early phase of RA disease development, showing RFs as the first-appearing antibodies, particularly of the IgA isotype with important implications for the understanding of breaking of tolerance.

## Additional files

Additional file 1: Table S1. Prevalence, sensitivity, specificity and OR of numbers of RF isotypes, alone and/or in combination with ten different ACPA specificities, anti-CCP2 antibodies and anti-carbamylated protein antibodies in pre-symptomatic individuals and population controls. (DOCX 17kb) Additional file 2: Figure S1. Illustration of a conditional inference tree for developing RA in pre-symptomatic individuals compared with control subjects. *CCP2* anti-CCP2 antibodies, *Filaggrin* anti-filaggrin antibodies, *CEP-1* = anti-α-enolase antibodies, *IgM-RF* immunoglobulin M rheumatoid factor, *HLA-SE* human leukocyte antigen shared epitope, *n* number of individuals, *Pr* probability of detecting a pre-symptomatic individual, + = positive, − = negative. (DOCX 29 kb)

## Abbreviations

ACPA: anti-citrullinated protein antibodies
AU: arbitrary units
anti-CarP: antibodies against carbamylated proteins
anti-CCP: anti-cyclic citrullinated peptide antibodies
Car: carbamylated
CEP-1: α-enolase
CI: confidence interval
CII: collagen type II
EV: explanatory variable
FCS: foetal calf serum
Fib: fibrinogen
HLA-SE: human leucocyte antigen–shared epitope
IgA: immunoglobulin A
IgG: immunoglobulin G
IgM: immunoglobulin M
IQR: interquartile range
OR: odds ratio
NPV: negative predictive value
PPV: positive predictive value
RA: rheumatoid arthritis
RF: rheumatoid factor
ROC: receiver operating characteristic
Vim: vimentin
