# Supplementary material for: Rheumatoid factor isotypes in relation to antibodies against citrullinated peptides and carbamylated proteins before the onset of rheumatoid arthritis
Source: Arthritis Res Ther. 2016 Feb 9;18:43. doi: 10.1186/s13075-016-0940-2 (PMC4748586; doi:10.1186/s13075-016-0940-2)
Supplement: Additional file 1: Table S1. — Prevalence, sensitivity, specificity and OR of numbers of RF isotypes, alone and/or in combination with ten different ACPA specificities, anti-CCP2 antibodies and anti-carbamylated protein antibodies in pre-symptomatic individuals and population controls. (DOCX 17kb) [file 13075_2016_940_MOESM1_ESM.docx]

#### Supplementary table 1. Prevalence, sensitivity, specificity and OR of numbers of RF isotypes, alone and/or in combination with ten different ACPA specificities, anti-CCP2 antibodies and anti-carbamylated protein antibodies in pre-symptomatic individuals and population controls.

| **Antibody/combination** | **Pre-symptomatic n** | **Controls n** | **Sensitivity % (95%CI)** | **Specificity % (95%CI)** | **OR (95%CI)** |
| --- | --- | --- | --- | --- | --- |
| ≥1RF | 237/598 | 47/492 | 39.6 (35.8-43.6) | 90.4 (87.5-92.7) | 6.2 (4.4-8.7) |
| ≥2RF | 114/598 | 8/492 | 19.1 (16.1-22.4) | 98.4 (96.7-99.2) | 14.3 (7-28.9) |
| CCP2 and/or ≥1RF | 290/598 | 54/492 | 48.5 (44.5-52.5) | 89 (85.9-91.5) | 7.6 (5.5-10.6) |
| CCP2 and/or ≥1RF and/or CarP | 226/421 | 24/150 | 53.7 (48.9-58.4) | 84 (77.2-89) | 6.1 (3.8-9.8) |
| CCP2 and/or ≥2RF | 208/598 | 16/492 | 34.8 (31.1-38.7) | 96.7 (94.7-98) | 15.9 (9.4-26.7) |
| CCP2 and/or ≥2RF and/or CarP | 163/421 | 6/150 | 38.7 (34.2-43.5) | 96 (91.3-98.3) | 15.2 (6.7-34.1) |
| CEP1 and/or ≥1RF | 275/590 | 70/484 | 46.6 (42.6-50.6) | 85.5 (82.1-88.4) | 5.2 (3.8-7) |
| CEP1 and/or ≥1RF and/or CarP | 215/414 | 25/147 | 51.9 (47.1-56.7) | 83 (76-88.2) | 5.3 (3.3-8.4) |
| CEP1 and/or ≥2RF | 188/590 | 35/484 | 31.9 (28.2-35.7) | 92.8 (90.1-94.8) | 6 (4.1-8.8) |
| CEP1 and/or ≥2RF and/or CarP | 164/414 | 14/147 | 39.6 (35-44.4) | 90.5 (84.5-94.3) | 6.2 (3.5-11.1) |
| CIIC1cit and/or ≥1RF | 257/590 | 62/484 | 43.6 (39.6-47.6) | 87.2 (83.9-89.9) | 5.3 (3.8-7.2) |
| CIIC1cit and/or ≥1RF and/or CarP | 199/414 | 27/147 | 48.1 (43.3-52.9) | 81.6 (74.5-87.1) | 4.1 (2.6-6.5) |
| CIIC1cit and/or ≥2RF | 152/590 | 26/484 | 25.8 (22.4-29.5) | 94.6 (92.2-96.3) | 6.1 (4-9.4) |
| CIIC1cit and/or ≥2RF and/or CarP | 137/414 | 14/147 | 33.1 (28.7-37.8) | 90.5 (84.5-94.3) | 4.7 (2.6-8.4) |
| Fibβ36-52 and/or ≥1RF | 272/590 | 73/484 | 46.1 (42.1-50.1) | 84.9 (81.4-87.8) | 4.8 (3.6-6.5) |
| Fibβ36-52 and/or ≥1RF and/or CarP | 213/414 | 31/147 | 51.4 (46.6-56.2) | 78.9 (71.6-84.7) | 4 (2.6-6.1) |
| Fibβ36-52 and/or ≥2RF | 187/590 | 39/484 | 31.7 (28.1-35.6) | 91.9 (89.1-94.1) | 5.3 (3.7-7.7) |
| Fibβ36-52 and/or ≥2RF and/or CarP | 163/414 | 19/147 | 39.4 (34.8-44.2) | 87.1 (80.6-91.6) | 4.4 (2.6-7.3) |
| Fibα563-583 and/or ≥1RF | 248/590 | 67/484 | 42 (38.1-46.1) | 86.2 (82.8-89) | 4.5 (3.3-6.1) |
| Fibα563-583 and/or ≥1RF and/or CarP | 196/414 | 27/147 | 47.3 (42.6-52.2) | 81.6 (74.5-87.1) | 4 (2.5-6.3) |
| Fibα563-583 and/or ≥2RF | 134/590 | 30/484 | 22.7 (19.5-26.3) | 93.8 (91.2-95.6) | 4.4 (2.9-6.7) |
| Fibα563-583 and/or ≥2RF and/or CarP | 126/414 | 14/147 | 30.4 (26.2-35) | 90.5 (84.5-94.3) | 4.2 (2.3-7.4) |
| Fibα580-600 and/or ≥1RF | 249/590 | 72/484 | 42.2 (38.3-46.2) | 85.1 (81.7-88) | 4.2 (3.1-5.6) |
| Fibα580-600 and/or ≥1RF and/or CarP | 193/414 | 25/147 | 46.6 (41.9-51.4) | 83 (76-88.2) | 4.3 (2.7-6.8) |
| Fibα580-600 and/or ≥2RF | 136/590 | 38/484 | 23.1 (19.8-26.6) | 92.1 (89.4-94.2) | 3.5 (2.4-5.1) |
| Fibα580-600 and/or ≥2RF and/or CarP | 125/414 | 14/147 | 30.2 (26-34.8) | 90.5 (84.5-94.3) | 4.1 (2.3-7.3) |
| Fibβ62-81a and/or ≥1RF | 257/590 | 60/484 | 43.6 (39.6-47.6) | 87.6 (84.3-90.2) | 5.5 (4-7.5) |
| Fibβ62-81a and/or ≥1RF and/or CarP | 202/414 | 24/147 | 48.8 (44-53.6) | 83.7 (76.8-88.8) | 4.9 (3-7.8) |
| Fibβ62-81a and/or ≥2RF | 146/590 | 23/484 | 24.7 (21.4-28.4) | 95.2 (92.9-96.8) | 6.6 (4.2-10.4) |
| Fibβ62-81a and/or ≥2RF and/or CarP | 136/414 | 12/147 | 32.9 (28.5-37.5) | 91.8 (86.1-95.4) | 5.5 (3-10.2) |
| Fibβ62-81b and/or ≥1RF | 255/590 | 54/484 | 43.2 (39.3-47.3) | 88.8 (85.7-91.4) | 6.1 (4.4-8.4) |
| Fibβ62-81b and/or ≥1RF and/or CarP | 201/414 | 22/147 | 48.6 (43.8-53.4) | 85 (78.3-89.9) | 5.4 (3.3-8.7) |
| Fibβ62-81b and/or ≥2RF | 154/590 | 16/484 | 26.1 (22.7-29.8) | 96.7 (94.6-98) | 10.3 (6.1-17.4) |
| Fibβ62-81b and/or ≥2RF and/or CarP | 143/414 | 9/147 | 34.5 (30.1-39.3) | 93.9 (88.6-96.9) | 8.1 (4.1-16.1) |
| Filaggrin and/or ≥1RF | 274/590 | 57/484 | 46.4 (42.5-50.5) | 88.2 (85-90.8) | 6.5 (4.7-8.9) |
| Filaggrin and/or ≥1RF and/or CarP | 216/414 | 26/147 | 52.2 (47.4-56.9) | 82.3 (75.3-87.7) | 5.1 (3.2-8.1) |
| Filaggrin and/or ≥2RF | 184/590 | 20/484 | 31.2 (27.6-35) | 95.9 (93.7-97.3) | 10.5 (6.5-16.9) |
| Filaggrin and/or ≥2RF and/or CarP | 164/414 | 13/147 | 39.6 (35-44.4) | 91.2 (85.3-94.8) | 6.8 (3.7-12.2) |
| Vim2-17 and/or ≥1RF | 244/590 | 63/484 | 41.4 (37.5-45.4) | 87 (83.7-89.7) | 4.7 (3.5-6.4) |
| Vim2-17 and/or ≥1RF and/or CarP | 191/414 | 25/147 | 46.1 (41.4-51) | 83 (76-88.2) | 4.2 (2.6-6.7) |
| Vim2-17 and/or ≥2RF | 130/590 | 29/484 | 22 (18.9-25.6) | 94 (91.5-95.8) | 4.4 (2.9-6.7) |
| Vim2-17 and/or ≥2RF and/or CarP | 122/414 | 13/147 | 29.5 (25.3-34) | 91.2 (85.3-94.8) | 4.3 (2.4-7.8) |
| Vim60-75 and/or ≥1RF | 252/590 | 67/484 | 42.7 (38.8-46.7) | 86.2 (82.8-89) | 4.6 (3.4-6.3) |
| Vim60-75 and/or ≥1RF and/or CarP | 193/414 | 27/147 | 46.6 (41.9-51.4) | 81.6 (74.5-87.1) | 3.9 (2.5-6.1) |
| Vim60-75 and/or ≥2RF | 145/590 | 30/484 | 24.6 (21.3-28.2) | 93.8 (91.2-95.6) | 4.9 (3.3-7.4) |
| Vim60-75 and/or ≥2RF and/or CarP | 129/414 | 15/147 | 31.2 (26.9-35.8) | 89.8 (83.7-93.8) | 4 (2.3-7) |
