# Supplementary figures and images for: Rheumatoid factor isotypes in relation to antibodies against citrullinated peptides and carbamylated proteins before the onset of rheumatoid arthritis
Source: Arthritis Res Ther. 2016 Feb 9;18:43. doi: 10.1186/s13075-016-0940-2 (PMC4748586; doi:10.1186/s13075-016-0940-2)

Figure S1


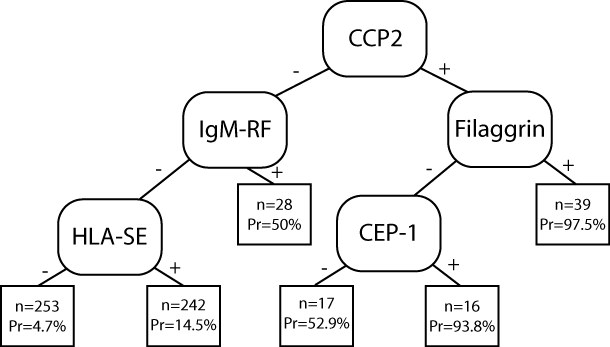

Supplement: Additional file 2: Figure S1. — Illustration of a conditional inference tree for developing RA in pre-symptomatic individuals compared with control subjects. CCP2 anti-CCP2 antibodies, Filaggrin anti-filaggrin antibodies, CEP-1 = anti-α-enolase antibodies, IgM-RF immunoglobulin M rheumatoid factor, HLA-SE human leukocyte antigen shared epitope, n number of individuals, Pr probability of detecting a pre-symptomatic individual, + = positive, − = negative. (DOCX 29 kb) [file 13075_2016_940_MOESM2_ESM.docx]
